# Research Progress on Nutritional Value, Preservation and Processing of Fish—A Review

**DOI:** 10.3390/foods11223669

**Published:** 2022-11-16

**Authors:** Ahtisham Ali, Shuai Wei, Adnan Ali, Imran Khan, Qinxiu Sun, Qiuyu Xia, Zefu Wang, Zongyuan Han, Yang Liu, Shucheng Liu

**Affiliations:** 1 College of Food Science and Technology, Guangdong Ocean University, Guangdong Provincial Key Laboratory of Aquatic Products Processing and Safety, Guangdong Province Engineering Laboratory for Marine Biological Products, Key Laboratory of Advanced Processing of Aquatic Product of Guangdong Higher Education Institute, Guangdong Provincial Engineering Technology Research Centre of Seafood, Zhanjiang 524088, China; 2 Livestock & Dairy Development Department, Abbottabad 22080, Pakistan; 3 Department of Food Science and Technology, The University of Haripur, Haripur 22620, Pakistan; 4 Collaborative Innovation Centre of Seafood Deep Processing, Dalian Polytechnic University, Dalian 116034, China

**Keywords:** fish, nutrition, preservation, processing, by-products

## Abstract

The global population has rapidly expanded in the last few decades and is continuing to increase at a rapid pace. To meet this growing food demand fish is considered a balanced food source due to their high nutritious value and low cost. Fish are rich in well-balanced nutrients, a good source of polyunsaturated fatty acids and impose various health benefits. Furthermore, the most commonly used preservation technologies including cooling, freezing, super-chilling and chemical preservatives are discussed, which could prolong the shelf life. Non-thermal technologies such as pulsed electric field (PEF), fluorescence spectroscopy, hyperspectral imaging technique (HSI) and high-pressure processing (HPP) are used over thermal techniques in marine food industries for processing of most economical fish products in such a way as to meet consumer demands with minimal quality damage. Many by-products are produced as a result of processing techniques, which have caused serious environmental pollution. Therefore, highly advanced technologies to utilize these by-products for high-value-added product preparation for various applications are required. This review provides updated information on the nutritional value of fish, focusing on their preservation technologies to inhibit spoilage, improve shelf life, retard microbial and oxidative degradation while extending the new applications of non-thermal technologies, as well as reconsidering the values of by-products to obtain bioactive compounds that can be used as functional ingredients in pharmaceutical, cosmetics and food processing industries.

## 1. Introduction

Fish is a widely cultivated food product with highly economical trading in Southeast Asian countries such as Hong Kong, Singapore, Malaysia and Thailand [1]. Fish production has been predicted to reach 196 million tons in 2025 worldwide [2]. Fish is a very diversified food commodity mostly cultured in tropical and subtropical regions. The demand for fish is significantly increasing with the increase in the world population because of their favourable taste, efficient feed conversion and high commercial value [3].

Fish are considered highly nutritious products of the aquaculture system due to the presence of well-balanced macronutrients such as proteins, lipids and micronutrients such as vitamins and minerals [4]. These fish are a good source of human food that promotes growth and protection of the body from a variety of health diseases such as cardiovascular and coronary heart diseases and prevents rickets and mental diseases in children [5]. The protein present in fish has high nutritional value because essential amino acids serve as antioxidant elements in various nutraceutical industries. These amino acids possess many properties such as gel formation, oil adsorption, water-holding capacity and health-related properties. Amino acids also have antihypertensive, blood quality maintenance, muscle tissue repairing and system-regulating properties in humans [6]. Lipids are important for health and are rich in polyunsaturated fatty acids (PUFAs), especially eicosapentaenoic acid (EPA) and docosahexaenoic acid (DHA) which help to prevent cardiovascular diseases and coronary heart diseases and maintain mental health in children [7]. Similarly, fish contains a perfect balance of all essential vitamins, especially vitamins A and D, and are also a significant source of vitamin B. Vitamin B mostly prevents calcium-deficient diseases and rickets in children. Minerals are micronutrients that vary from species to species, including calcium, iron, zinc, selenium, iodine, phosphorus and potassium. These micronutrients have high bioavailability and antioxidant properties that are useful for curing various diseases [8].Fish and their products can be spoiled easily if not preserved properly. Fish and fish product quality deteriorates because of digestive enzymes, lipid oxidation and microbes which actively contribute to fish spoilage [9]. Compositional changes in protein and lipids lead to the development of new products that cause physiological and chemical changes. Therefore, it is necessary to understand and minimize the factors that contribute to fish spoilage by using active preservation techniques to sustain the freshness of fish and fish-containing products [10]. Various preservation techniques are used to preserve and process fish at an industrial level such as pulsed electric field pulsed electric field (PEF), fluorescence spectroscopy, hyperspectral imaging technique (HSI) and high-pressure processing (HPP) while traditional techniques include cooling, freezing and super-chilling [11]. Excellent food preservation techniques effectively prevent microbial spoilage and prolong the product shelf life with limited adverse changes in the quality and nutritional values, texture and flavour. Many studies have focused on chemical and low-temperature storage methods for fish preservation. Fish is a part of a healthy diet and provides essential components such as proteins, vitamins, polyunsaturated fatty acids and minerals that are necessary for healthy growth. Fish is a highly perishable food and its quality is adversely affected during storage by several factors such as enzymatic autolysis, microbial growth and oxidation [12].

**Figure 1:**
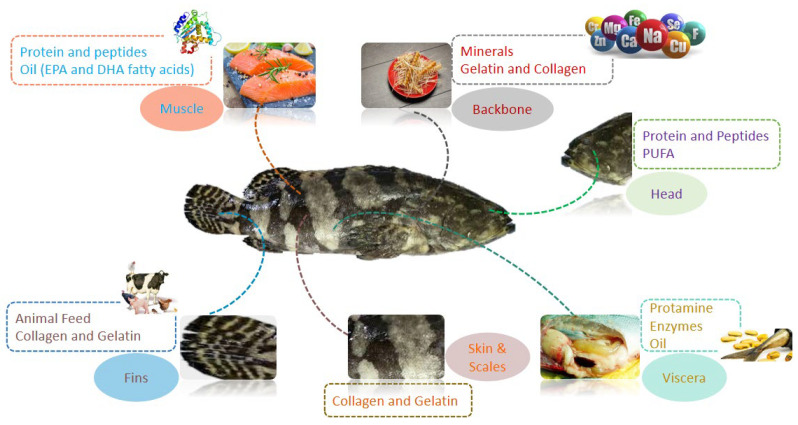
Bioactive compounds present in various parts of fish.

Extensive amounts of by-products are produced as a result of fish processing and are estimated to be up to 60% of the total fish weight [13]. Usually, fish processing by-products are dumped as waste in oceans and on land and contain highly valuable components that can cause serious environmental pollution. These by-products are also used as dietary components in fish meal, silage and fertilizer production. Fish processing by-products contain components such as the skin, scales, viscera, head, trimmings, roe and bones which are unfit for human consumption and are discarded as waste [14]. These by-products are a good source of nutritional components, especially lipids and proteins as well as functional components (Figure 1).

New processing technologies are being used to facilitate the production of highly valuable marketed products that can obtain high economic prices. In this way, discarded waste can be reduced and environmental pollution can also be reduced. Therefore, it is currently considered a necessary and challenging factor to develop new technologies to enable the recovery of valuable fish processing by-products for obtaining functional ingredients that can be used as high-value-added products for human consumption [15].

The objective of this current review is to revise the importance of the nutritional value of fish and focus on the potential applications of preservation technologies including low-temperature-based techniques and antimicrobial and antioxidant preservatives. This review article also summarizes the possible applications of thermal and non-thermal processing technologies as well as the production and utilization of various bioactive compounds at an industrial level.

## 2. Nutritional Value of Fish

Fish are among the most commercially valuable species in Asia. Moreover, fish are considered key species in coastal ecosystems, and their decline due to fishing pressure has a significant impact on the ecosystem. Therefore, overfishing to meet market demand is a concern [16]. Furthermore, the nutritional value of fish has shown some beneficial effects on human health with efficient protective measures against cardiovascular diseases, cancer and Alzheimer’s disease [17]. Fish has contained high nutritional value due to having rich contents of protein, water, amino acid composition and fatty acids [18].

### 2.1. Proteins

Fish protein has long been considered to have a high nutritional value due to its being rich in many bioactive peptides and essential amino acids. They are readily digested due to the presence of low connective tissues and can be used for various metabolic activities [19]. These proteins have various pharmaceutical and nutraceutical applications and are being efficiently used as functional ingredients in many food items. Even though they have some useful properties such as oil absorption, water-holding capacity, gel formation, emulsification and foaming properties [20]. In addition, fish protein has various significant bioactive properties such as antioxidative, antithrombic and antihypertensive properties (Table 1). Fish proteins are used to repair muscle tissues, and improve immunity and blood quality. Fish proteins can also be used to prevent protein–calorie malnutrition (PCM) in animals [21].

In addition to being a food source, protein also performs various dominant functions to prevent bacterial and viral infections and helps to maintain the water balance and regulatory system in the human body [29]. The amino acids of proteins have a variety of nutritional values, chemical actions and medicinal properties. For instance, amino acids are used in pharmaceuticals as an excipient for drug development and employed as a food additive in food and feed supplement sources. In the flavouring industry, amino acids such as alanine, aspartate, monosodium glutamate and arginine are the most commonly used flavour enhancer ingredients in a variety of foods. Amino acids have various applications in the pharmaceutical industry such as purifying proteins and are used in the formulation and production of many antibiotics [30].

### 2.2. Lipids

Lipids play an important role in the nutritional value of fish due to the presence of long-chain PUFAs which consist of omega-3 fatty acids, particularly EPA and DHA [26]. These fatty acids have great beneficial impacts on human health and nutrition and prevent various diseases [31]. PUFAs help to reduce blood pressure and high concentrations of triglycerides in blood vessels. The high intake of fatty acids proved to have a beneficial impact on preventing cardiovascular diseases. Omega-3 fatty acids are mostly recommended as an essential element in the growth of children and have some preventive effects against coronary heart diseases [32].

Among fatty acids, DHA is particularly good for optimizing brain growth and neurodevelopment in children while EPA is important for cardiovascular health [33]. Many other benefits include prevention against arrhythmias, therapeutics for asthma patients, protection against atherosclerosis and manic-depressive illness, reduced symptoms of cystic fibrosis and survival of cancer patients [34]. The American Heart Association has recommended at least two servings of fish per week to reduce the risk of cardiovascular diseases. In addition, these fatty acids are used in biodiesel production through enzymatic transesterification of fish oil. This type of biodiesel has become a newly trending nontoxic, biodegradable and renewable energy source [35].

### 2.3. Multi-Vitamins

Fish also contain the perfect balance of all essential vitamins which play an important role in human health. Fish is a rich source of vitamins (A and D) and a good source of B-group vitamins which are considered to be beneficial for the growth and development of children [36]. Vitamin A maintains cell development, the formation of bones and teeth and it also significantly contributes to improving weak eyesight as well as the treatment of various eye-related diseases [37]. Vitamin D present in fish was found in the form of vitamin D3 (cholecalciferol) which represents a three-fold higher potential efficiency ratio than that of vitamin D (ergocalciferol) and it was also found in the skin as 7-dehydrocholesterol after exposure to ultraviolet light [38]. Most children suffer from vitamin D deficiency that causes rickets but it is also found common in adults where many other diseases such as osteoporosis, osteomalacia, osteopenia, low bone mineral density and diabetes have been reported [39]. Vitamin B accelerates enzyme functioning which facilitates chemical processes in the human body whereas vitamin K is important for blood coagulation and helps to prevent internal bleeding in the body [8].

### 2.4. Minerals

Most micronutrients with high bioavailability are present in fish within the range of approximately 0.4 to 1.5%. Fish contain high-nutritional-value minerals in widely varying quantities including calcium, iron, zinc, selenium, iodine, phosphorus and potassium [40].

In particular, iodine and selenium are considered to have significant nutritional value due to their high bioavailability. Iodine is essential for hormone production, especially thyroxin which helps to regulate the body’s metabolism. It is also important for the psychological and growth development of children. Selenium possesses some antioxidant properties and is an important micronutrient in the human body that only performs various functions in the form of selenoproteins. These proteins are directly responsible for normal thyroid function and the inactivation of antioxidant enzymes such as glutathione peroxidase [41]. Calcium is significantly used for bone formation and mineralization, and the proper functioning of muscles and the nervous system [42]. Iron is directly involved in the synthesis of haemoglobin in red blood cells (RBCs) that can assist in the regulation of oxygen in every body part [43].

## 3. Preservation Technology Approaches of Fish

Fish are considered a most highly perishable food commodity. Fish spoilage can be caused by the following three reasons: enzymatic autolysis, microbial deterioration and chemical activities. Deterioration mainly caused by chemical and microbial activities has annually contributed approximately 25% of gross primary agricultural and fishery product losses [44]. Microbial activity contributed to one-fourth of the worldwide food supply losses and 30% of fish product losses. Recently, fish preservation techniques have gained increasing interest because the prevention of microbial spoilage of fish without adversely affecting its nutritional quality, prolonged shelf life, flavour and textural quality has also been improved [45].

### 3.1. Low-Temperature Preservation

**Figure 2:**
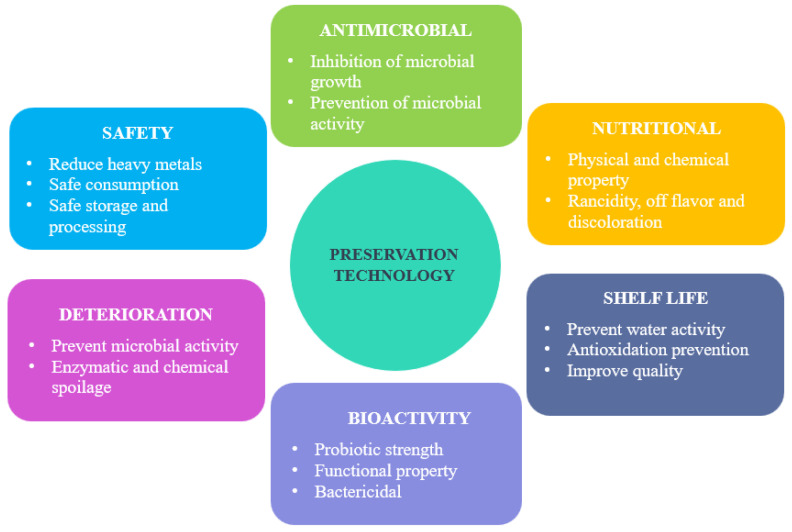
Advantages of various resulting factors obtained from preservation technology of fish.

Fish and fish-based products are usually used to preserved by various techniques at a very low temperature. There are several techniques such as cooling, freezing, icing and super-chilling that have been used to improve the preservation quality of fish [46] (Figure 2).

#### 3.1.1. Cooling and Icing Technique

Fish handled at high temperatures is more susceptible to fish spoilage than that handled at low temperatures [47]. Cooling renders microbial growth at −1 °C to 4 °C and freezing requires temperatures of about −18 °C to −30 °C to inhibit bacterial growth but both enzymatic and non-enzymatic changes continue at a minimal rate [48]. Icing is used to store fish at high temperatures before using the freezing method but the rate of deterioration depends upon the harvesting and handling approach. The icing technique has been used to perform different functions including maintenance of uniform low temperatures, reducing autolysis and bacterial degradation and providing gentle washing and cleaning during melting [49]. In a cooling technique, both enzymatic and non-enzymatic activities remain and continue with microbial growth. Therefore, the freezing method is used to control microbial growth as well as enzymatic activity [50].

#### 3.1.2. Freezing Technique

Freezing is the most efficient method used to control microorganisms and slow down the chemical changes that occur during storage. Fish contains 60–80% water that is converted into ice during the freezing process [3]. Freezing is a very fast process in which approximately 90–95% of water freezes at a temperature of −25 °C (75% of the water freezes at −5 °C in fish muscle). Approximately 10% of water remains unfrozen due to hydrogen bonding and chemically bonded water at a specific site such as the carbonyl and amino groups of protein [51]. The quality of frozen fish is significantly influenced by freezing time, i.e., fast and slow freezing. Freezing is a thermophysical phenomenon where fish are stored at low temperatures that affect fish quality due to varied ice crystal formation. Fast freezing (small ice crystals) produces better frozen fish quality than slow freezing (large ice crystals). Slow freezing causes tissue damage and denaturation of proteins [52].

During the freezing process, physical, chemical and biochemical reactions remain continuous in frozen fish and do not stop after cold treatment [53]. At a temperature of −12 °C, microorganism growth is minimal; below −18 °C, cellular metabolism is inhibited and at −55 °C quality changes are minimized [54]. Nevertheless, enzymatic and oxidative damage and ice crystallization lead to fish spoilage [55].

#### 3.1.3. Super-Chilling

Super-chilling is another low-temperature preservation technology used to keep the fish between chilling and freezing temperatures. The super-chilling process keeps fish below their initial freezing point (1–2 °C) [56]. This technique is considered to be different from the cooling and freezing methods and has the ability to minimize storage and transport costs. In this process, ice is added inside the fish muscles to freeze internal water which then acts as a refrigeration source during transportation and distribution [3]. Super-chilling extends the shelf life of fish at least 1.4–5 times that make it a more promising technique than most traditional methods [57]. Super-chilling temperatures are used to reduce microbial growth and bacterial activity but physical and chemical changes may occur [58].

### 3.2. Antimicrobial Preservation

**Figure 3:**
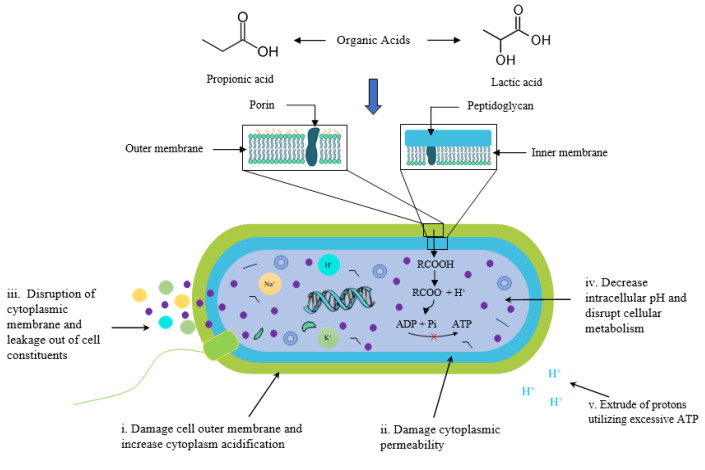
Antimicrobial mechanism of organic acids in a fish bacterial cell. ATP: Adenosine triphosphate; ADP: Adenosine diphosphate.

Several antimicrobial compounds including nitrites, sulfites and organic acids are used to control the microbial load [59] (Table 2). Nitrites along with sodium chloride are generally used as salt reservoirs (sodium nitrite and potassium nitrite) in fish products as antimicrobial agents for toxin-producing compounds (*Clostridium botulinum*) and used to improve colour [60]. Nitrites adversely affect the activity of microorganisms through several reactions including (a) reacting with an alpha-amino group of amino acids at low pH, (b) blocking the sulfhydryl group, (c) reacting with iron-containing compounds that inhibit the bacteria from using iron and (d) interfering with membrane permeability which limits transport across cells [61] (Figure 3).

**Table 2:** Application of natural antimicrobial and antioxidant preservatives from different fish and fishery products.

Antimicrobial	Preservatives	Fish/Fish Products	Storage Life	Main Effects	Reference
Plant origin	Wild mint leaf and cumin seed	Rainbow trout muscle	Up to 12–18 days	Total viable count and psychrotrophic bacteria ↓, peroxide value ↓, thiobarbituric acid reactive substances ↓, lipid oxidation ↓, sensory quality ↑	[63]
	Pure lemon essential oil (*p*-cymene 14.36%, D-limonene 52.85% and β-pinene 13.69%)	Fish spoilage bacteria	Shelf life increased	*Photobacterium damselae* ↓, *Vibrio vulnificus* ↓, *Proteus Mirabilis* ↓, *Serratia liquefaciens* ↓, *Enterococcus faecalis* ↓, *Pseudomonas luteola* ↓, shelf life ↑	[64]
	Cinnamon oil (Immersion 0.1%)	Common carp muscle	Up to 2 days	Total volatile base nitrogen ↓, total viable count ↓, biogenic amines ↓, H_2_S producing bacteria ↓, lactic acid bacteria ↓, *Pseudomonas and Aeromonas* ↓, shelf life ↑, sensory quality ↑	[65]
	*Salvia officinalis L.*	Rainbow trout muscle	Shelf life increased (up to 25 days)	Total mesophilic count, *Pseudomonas*, *Enterobacteriaceae*, psychrophilic and H_2_S producing bacteria, formation of total volatile base nitrogen and free fatty acid ↓, shelf life ↑	[66]
	Marinated crayfish (Immersion 30 mL/L)	Rosemary and thyme	Up to 42–70 days	Thiobarbituric acid value ↓, total viable count ↓, total volatile basic nitrogen ↓, psychrotrophic bacteria count ↓, lactic acid bacteria ↓, moulds and yeast ↓, sensory score and shelf life ↑	[67]
Animal origin	Chitosan and lysozyme (Immersion 0.6 mg/mL)	Large yellow croaker	Shelf life increased (up to 15 days)	Lipid oxidation ↓, thiobarbituric acid value ↓, total volatile basic nitrogen ↓, total viable count ↓ (7.0 log CFU/g), *Salmonella*, *S. aureus, E. coli*, *P. aeruginosa* ↓, shelf life ↑	[68]
	Chitosan and glycerol monolaurate (0.1% and 0.3%)	Grass carp	Up to 15–20 days	Total viable count ↓, psychrophilic bacteria counts ↓, *pseudomonads* ↓, H_2_S producing bacteria, thiobarbituric acid value ↓, total volatile basic nitrogen ↓, shelf life ↑	[69]
	Pomegranate peel extract-lysozyme, gelatin	Mackerel	Shelf life increased (up to 9 days)	Mesophilic and psychrotrophic count ↓, bacterial activity ↓, free fatty acids and thiobarbituric acid reactive substances ↓, sensory and shelf life ↑	[70]
	Lactoperoxidase and whey	Rainbow trout muscles	Up to 12–16 days	*Mesophiles*, *S. putrefaciens*, *pseudomonas* spp., *P. fluorescens* ↓, sensory quality and shelf life ↑	[71]
Bacteriocins	Lactic acid bacteria and essential oil	Sea bass	14–21 days	Psychrotrophic bacterial count ↓, mesophilic aerobic plate count ↓, total volatile basic nitrogen ↓, shelf life ↑	[72]
	Bacteriocin 7293	Pangasius fish fillets	Up to 6–7 days	Gram-positive (*S. aureus* and *L. monocytogenes*) ↓, Gram-negative (*A. hydrophila, S. typhimurium*, *P. aeruginosa* and *E. coli*) bacteria	[73]
	Reuterin isolated by *Lactobacillus reuteri INIA P579*	Cold smoked salmon	Shelf life increased (up to 15 days)	*E. coli K12* ↓, *L. monocytogenes strains* ↓, pathogenic bacterial growth ↓, shelf life ↑	[74]
	Bacteriocin EFL4	Fresh salmon fillets	Shelf life increased (up to 7 days)	*S. aureus, E. coli, S. putrefaciens, P. fluorescens* and *L. monocytogenes* ↓, total viable count ↓, total volatile basic nitrogen ↓, shelf life ↑	[75]
Organic acids	Citric and lactic acid	European lake	Shelf life increased (up to 15 days)	Aerobe and anaerobe ↓, psychrotrophic and *enterobacteriaceae* counts ↓, proteolytic activity ↓, sensory quality ↑	[76]
	Citric and acetic acid (1 and 3%)	Bolti fish	Shelf life increased (up to 12 days)	Viable bacterial count ↓, coliform, yeast and mould count ↓, psychrophilic bacteria ↓, microbial load ↓	[77]
	Acetic and ascorbic acid	Silver carp	Shelf life increased (up to 9 days)	Total viable count ↓, peroxide value and pH ↓, bacterial activity ↓, sensory quality ↑, shelf life ↑	[78]
	Sodium lactate, sodium acetate, sodium citrate	Salmon	Shelf life extended (4–7 days)	Aerobic and psychrotrophic count ↓, *Pseudomonas spp.*, lactic acid and *Enterobacteriaceae* bacteria ↓, H_2_S producing bacteria ↓, shelf life ↑	[79]
Extract type	Nisin	Rainbow trout	Shelf life extended (12–16 days)	Lipid oxidation ↓, total viable count ↓, psychrotrophic viable count ↓, bacteriostatic action ↓, total viable count ↓, peroxide value ↓, thiobarbituric acid value ↓, shelf life ↑	[80]
	Rosemary extract with nisin stored at 4 °C	Golden pompano fillet	Up to 6 days	Protein degradation ↓, nucleotide breakdown ↓, microbial count and lipid oxidation ↓, total volatile basic nitrogen ↓, colour, texture and sensory attributes ↑, shelf life ↑	[81]
	*Urtica dioica* extract with whey protein contained poly (ε-caprolactone)	Rainbow trout fillet	Up to 15 days	Antimicrobial and antioxidant activity ↑, bacterial growth ↓, total volatile basic nitrogen and thiobarbituric acid values ↓, inhibition against mesophilic, psychrophilic, lactic acid bacteria and *enterobacteriaceae* ↑	[82]
	*Lactobacillus reuteri* combined with modified atmosphere packaging	Tuna burger	Up to 12–13 days	Colour, odour and juiciness ↑, microbial quality ↑, product quality ↑, shelf life ↑	[83]
Antioxidants	Phenols	Salted silver carp	Up to 6 days	Oxidative stability ↑, thiobarbituric acid reactive substances ↓, lipoxygenase ↓, sensory quality ↑	[84]
	Ginger extract supercritical and essential oil (*β sesquiphellandrene*, *α-Zingiberene*, *β-bisabolene*, *α-farnesene*, *α-curcumene*)	Nile tilapia burger	Up to 6–8 days	Thiobarbituric acid reactive substances ↓, antioxidant activity ↑, lipid oxidation ↓ reduced by enzymatic activities (*Catalases-CAT, Total superoxide dismutase-SOD and Glutathione peroxidase-GSH-px*), shelf life ↑	[85]
	Chitosan with essential oil (*clove bud*, *cinnamon* and *lemongrass*)	Grass carp fillets	Up to 7–11 days	Deterioration of physicochemical quality ↓, microbial growth ↓, oxidative stress ↓, lipid oxidation ↓, shelf life ↑	[86]
	Nisin coated with chitosan	Yellow croaker	Shelf life extended (6–9 days)	Microbial growth ↓, lipid oxidation ↓, protein oxidation ↓, shelf life ↑	[87]
	Nisin combination with high-pressure processing (450 and 600 MPa) at low temperature (−3 °C)	Dry-cured cold smoked salmon	Shelf life increased	*Listeria* spp. ↓, spoilage microbiota ↓, sensory quality and peelability ↑, consumer preference ↑	[88]
	Satureja thymbra extract (*ɣ terpinene*, *p-cymene*, *carvacrol* and *trans-caryophyllene*)	Gilthead seabream	Shelf life extended (25–35%)	Lipid oxidation (peroxide value) ↓, antimicrobial activity ↓, shelf life ↑	[89]
	Oregano essential oil	Hake burgers	Shelf life increased (up to 14 days)	bacterial count ↓, lipid oxidation ↓, shelf life ↑	[90]
	*Halocnemum strobilaceum* (Phenolic content 500 mg GAE/L)	Dolphinfish (Coryphaena hippurus) fillet	Shelf life increased (up to 6–9 days)	Lipid oxidation ↓, peroxide value ↓, malondialdehyde ↓, sensory properties ↑, shelf life ↑	[91]
	Chitosan (2% w/v) and nano-chitosan (2% w/v)	Silver carp	Shelf life increased (3–6 days)	Antimicrobial activity ↓, total volatile basic nitrogen ↓, lipid oxidation ↓, thiobarbituric acid value ↓, mesophilic and psychrophilic bacteria count ↓	[92]
	Chitosan oligosaccharides (COS)—nisin conjugates	*Collichthys niveatus*	Shelf life increased (4 days)	Sensory and texture deterioration ↓, total viable counts ↓, total volatile basic nitrogen ↓, oxidative spoilage ↓, shelf life ↑	[93]

↓ Decrease or inhibit, ↑ Increase.

Sodium chloride is commonly used in fish preservation at high concentrations and it is significantly used to inhibit microbial growth by increasing osmotic pressure and decreasing water activity. A combination of both sodium chloride and sodium lactate has a great impact on the quality, colour, fat stability and overall spoilage of fish [62].

Sodium sulphites have antimicrobial activity against aerobic, Gram-negative bacilli, yeasts and moulds of fish and fish products. Sulphurous acid enters the cell and reacts with thiol groups of enzymes, proteins and cofactors [94]. Sulphite blocks cystine disulfide linkages because it reacts with cellular adenosine triphosphate (ATP) leading to yeast death [95].

Lactic acid is used as an antimicrobial agent against pathogenic microorganisms (*Clostridium botulinum*) due to a reduction in the pH level and transfer of protons across the cell membrane [96]. Lactic acid bacteria serve as the inoculum in a newly developed method for fish preservation. These bacteria are very efficient in removing undesirable microorganisms and species of psychrotrophic lactic acid bacteria by producing lactic acid and other organic acids [97].

### 3.3. Antioxidant Preservation

**Figure 4:**
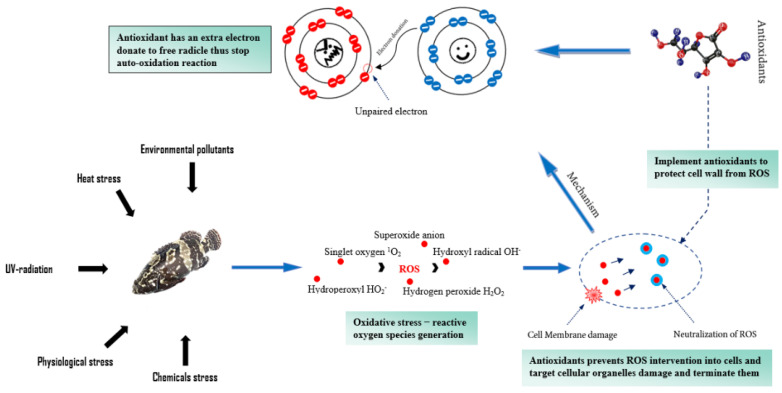
Mechanism of action of antioxidants against oxidation prevention. ROS stands for reactive oxygen species.

Oxidative deterioration in fish is mainly caused by the production of rancidity, off-flavours, discolouration and free radical catalyst due to the availability of oxygen in protein and lipid compounds [98]. Therefore, it is necessary to minimize the oxidation activity in fish products by using various antioxidant compounds (Table 2). Among them, the most commonly used lipid oxidation inhibitory additives include phenolic antioxidants (primary antioxidants) and phosphatase (a secondary antioxidant) [99]. Butylated hydroxyanisole (BHA) and ethylenediaminetetraacetic acid (EDTA) are considered to be derivatives of phenols that work as synthetic phenolic antioxidants. These antioxidants are widely used to dismiss chain-carrying peroxyl radicals and to inhibit the production of primary radicals and secondary radicals of lipids mainly to prevent, delay or inhibit the adverse effects of lipid peroxidation [100] (Figure 4).

Ethylenediaminetetraacetic acid (EDTA) is known as a sequestering agent, and is widely investigated for its ability to act as an antioxidant, chelating and metal complexing agent [101]. EDTA is a derivative of the polyaminocarboxylic acid group, added to fish to perform critical functions such as (a) acting as a pro-oxidant for the removal of trace metals through chelation, (b) acting as an inhibitor of lipid oxidation, (c) having antimicrobial activity by binding the divalent cations found in bacterial cell walls, (d) suppressing the growth of *Pseudomonas* species and (e) recovering the functional proteins by an acid solubilization process [102].

## 4. Processing Technology Approaches of Fish

A wide range of technologies is being used for the processing of commercially available fish products ranging from simple to advanced processing techniques including various treatments in order to meet consumer demands in terms of minimal processing and usage of chemical preservatives. However, the food industry is interested in developing non-thermal technologies including pulsed electric field (PEF), fluorescence spectroscopy, high-pressure processing (HPP) and thermal technologies such as canning.

### 4.1. Non-Thermal Technology

#### 4.1.1. Pulsed Electric Field (PEF)

The pulsed electric field (PEF) technique is used as a new emerging non-thermal processing technology based on electrical currents between two electrodes where short electrical pulses are employed with high voltages that enable the thermal effects to remain low [103]. Such characteristics make it different from other thermal electrical techniques such as ohmic heating [104] and moderate electrical fields [105].

PEF is predominantly used to reduce microbial activity, for extraction of value-added compounds, make improvements in the extraction of plant materials, enhancement of mass transfer through cell disruption and reduce the induction of stress in cells and damage the biological cells without paying any adverse effects on marine food [106]. PEF operates continuously in a very short time duration (milliseconds to microseconds) which makes it a more desirable processing technique than traditional methods. Most recently, PEF has been applied for the extraction of high-value-added components and to study mass transfer phenomena [107]. Concerning the extraction process, the high intensity of PEF has the potential to extract calcium [106] and chondroitin sulfate [108] with a high extraction efficiency ratio in a very short period of time. Likewise, abalone viscera protein with extensive emulsifying properties was extracted with a conventional extraction technique and the viscosity and foaming properties of most of the extracted product were decreased when PEF was applied [109].

#### 4.1.2. Fluorescence Spectroscopy

Fluorescence spectroscopy is known as a non-thermal processing technology since gaining interest due to efficient control of the quality and authenticity of fish and fish products [110]. Fluorescence spectroscopy is particularly applied to evaluate the freshness quality of most fish species stored under different light and vacuum packaging conditions and to monitor the access of lipid oxidation occurring in fatty and lean fish species during various storage and processing conditions such as canning, refrigerated and frozen storage [111]. The potential application of fluorescence spectroscopy was investigated to monitor the freshness quality among four groups of lean fish species; fish fillets stored for up to 12 days under various light and vacuum packaging conditions (dark/partial vacuum, dark total vacuum, light/partial vacuum and light/total vacuum) [111]. The findings of this study investigated that the fish fillets stored in dark and packed in a total vacuum had maximum quality characteristics compared to fillets kept in the other preservation conditions. Fluorescence spectroscopy has the potential to investigate the conformational changes that may occur in proteins as well as in the secondary and tertiary structures of proteins during processing [112]. In a recent study, [113] investigated the interaction between myofibrillar protein and lipids using Schiff structures, formed during cooking process as a result of protein oxidation in farmed sturgeon (*Acipenser gueldenstaedtii*). The emission spectra have shown various patterns of spectra after obtaining excitation set at 360 nm as a result of cooking methods. The fluorescence intensity obtained from cooking methods (frying and roasting) was much greater than the fluorescence intensity of other cooking methods of heated samples [113]. Collectively, particular focus is being placed on the investigation of this technique as a highly sensitive and selective approach compared to other traditional and spectroscopic methods due to possessing the characteristics of rapid and non-destructive detection of quality parameters, extension of shelf life and analysis of marine food products [114].

#### 4.1.3. Hyperspectral Imaging Technique (HSI)

Several spectroscopic techniques are used in fish and fish product analyses including near-infrared spectroscopy (NIR) and mid-infrared spectroscopy (MIR) [115], hyperspectral imaging (HSI) [116] and nuclear magnetic resonance spectroscopy (NMR) [117]. Recently, the hyperspectral imaging technique has been deployed as a rapid, non-destructive, smart and promising analytical tool to generate spatial and spectral information of the tested sample simultaneously [118]. Many recent reports have demonstrated the potential application of this technique to predict quality andauthenticity issues in fish and meat products such as microbial spoilage, texture quality, colour attributes and discrimination between fresh and frozen/thawed products [119].

HSI has been used to determine the fish freshness quality parameters such as total volatile basic nitrogen TVB-N and K-value and basic nutritional composition (moisture, crude protein, crude fat and fat-related compounds). For instance, hyperspectral imaging wavelength (308–105 nm) was employed to determine the K-value in grass and silver carp, as a result obtained a coefficient of determination for prediction (R^2^p = 0.94) [120]. To determine textural quality, Ma et al. [121] evaluated textural (hardness, chewiness and gumminess) parameters of vacuum freeze-dried fishfillets using hyperspectral imaging in the VIS-NIR region.

#### 4.1.4. High-Pressure Processing (HPP)

High-pressure processing (HPP) is another potential non-thermal processing technique progressively used to destroy microbial cells by disrupting noncovalent bonds, sustaining organoleptic properties and nutritional value without using high temperatures and increasing the shelf life of fish without compromising the sensory attributes [122].

In HPP, fish products are exposed to approximately 100–1000 MPa of pressure, fish products require very low temperatures (0–20 °C) to extend their shelf life and cannot be thermally processed while HPP allows for the application of high temperatures under cold conditions, resulting in an increased shelf life (by up to at least 300%) with reduced energy use and waste production [123]. Generally, HHP has extensively been utilized in the food processing industries in order to improve the nutritional quality of various kinds of food products such as dairy products, vegetables, fruits, including fish, meat and meat products, also including a variety of purposes such as prolonging the shelf lives, inhibit enzymatic and microbial degradation, modification in physicochemical properties, changes in microstructures and products development [103]. Greek national funds through the Operational Program “Competitiveness, Entrepreneurship and Innovation 2014–20” of the National Strategic Reference Framework (NSRF) (2016–19) investigated the effective application of HPP on fish physicochemical, microbiological and sensory attributes by assessing the quality and fingerprint recognition of microbial spoilage by using the “omics” technique. “Omics” technologies progressively identify and detect pathogens and bacteria to ensure the quality and safety of fish products by detecting the effect of protein-based processing in fish products [124].

### 4.2. Thermal Technology

#### Canning

Canning is the most widely used non-thermal technology for the preservation of fish over a prolonged period of time. Canned fish is typically processed at a specific heating temperature (113–160 °C) in a sealed airtight container for a specific time interval [124]. During the canning process, the product is processed in the centre of the can where the temperature increases slowly which affects the processed product for the shortest time duration [125].

Canned fish quality is based on the amino acid concentration, microbial activity, processing conditions and histamine concentration which play an important role in the investigation of fish quality. Appropriate handling of fish from harvesting to processing showed better initial fish quality with a great impact on final product quality and stability compared to insufficient handling. Additionally, *Bifurcaria bifurcate* extract was intentionally added to the packing of canned fish to enhance the product quality [126]. Heat treatment (a temperature of 90 °C) before canning led to fish with a lower liquid holding capacity and water content, resulting in a lower heating yield and a tough texture [127]. In thermal canning, safety parameters and prolonged shelf life are achieved by consuming a large amount of energy. Recently, many studies have investigated the progressive application of alternative energy sources in fish canning, among which thermo-solar heat pump sets are becoming popular in industrial processing [128].

In conclusion, traditional thermal processing methods provide acceptable information and well-tasting products. Despite this, they have many limitations such as long processing time, limited heat penetration and heterogeneous distribution of heat, which can lead to underheating or overheating problems [129]. Contrary to thermal technologies, non thermal technologies do not use direct heating as their main aim is to have minimal effects on the different quality parameters such as taste, texture, colour and aroma without changing the composition of nutritional components while achieving high-quality objectives and meeting consumer demands for minimally processed and without having any preservatives present in the food [130]. In contrast, non-thermal technologies showed greater advantages over traditional thermal technologies because of their reduce processing time, maximum extraction efficiency, nontoxicity, minimum solvent consumption and ecofriendliness [103]. In this regard, the advantages and disadvantages of non-thermal technologies are presented in Table 3.

## 5. Utilization of Fish Processing By-Products

**Figure 5:**
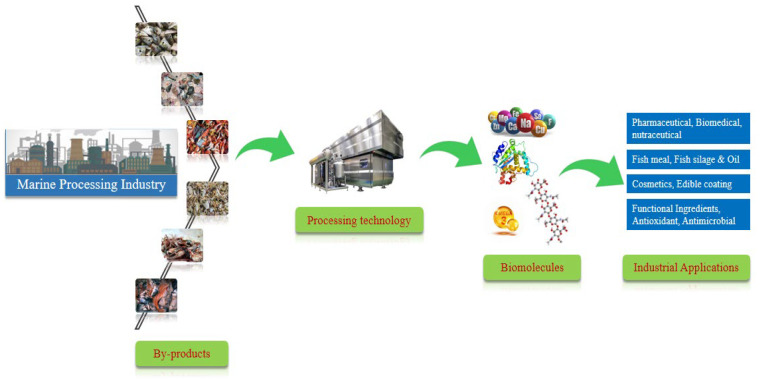
General processing steps involved in the extraction of bio-molecules and their various industrial applications.

Increasing fish waste production has become a serious issue worldwide over the last few decades. Technical, biological, operational and economic factors influence fish processing by-product production [141]. According to recent studies, total fish production leads to approximately 20% to 80% fish waste and unwanted products that have been discarded as waste depends on the species and applied processing methods [142]. In terms of industrial production, 40% of fish products are used for human consumption and the remaining 60% are waste products that include fins, viscera, head, skin and trimmings [13]. Therefore, the disposal of fish by-products is a serious problem in many regions and causes serious environmental pollution that could be overcome by using different techniques to utilize fish by-products [143] (Figure 5).

### 5.1. Fish Meal

Fish meal can be obtained by drying or grinding whole fish or fish by-products. Among fish species, mostly menhaden, capelin and anchovy fishes are used for fish meal production, which are the best sources of animal feed [144]. Chemically, fish meal is composed of minerals (10%), protein (70%), water (8%), fat (9%), vitamins, ash, pantothenic and various other minerals. Fish meal quality depends upon the types of amino acids, freshness and solubility of the fish meal and the processing method [145]. Fish meal is used as a feed for marine fish, crustaceans, poultry, ruminants, pigs and pets [146].

### 5.2. Fish Oil

Fish oil is derived from fish by-products and other unwanted products are also the main constituent of animal feed. Fish oil is composed of fatty acid triglycerides, phospholipids, glycerol ethers and wax esters that can remove environmental contaminants [147]. Numerous studies have been recently conducted on the extraction of fish oil by chemical and enzymatic transesterification because fish waste is a good source of lipids (60% by weight) [148]. Transesterification of fish oil and pyrolysis of fish oil is the process of recycling fish waste and producing char in the form of activated carbon and pyrolytic oil.

Fish oil is also suitable for biogas production, oil extraction and transformation to biodiesel [149]. During anaerobic digestion, a large amount of ammonia is released from fish waste, which greatly involves alleviating the level of methanogenic bacteria and a co-fermentation process is used to optimize the carbon-to-nitrogen ratio of a batch. In biorefinery industries, the co-fermentation process is conducted to recycle the energy material with the purpose of producing primary products including concentrated liquid material, secondary product-purified water, solid fertilizer and carbon dioxide [150].

### 5.3. Fish Silage

Fish waste is also utilized for the production of fish silage by the complete mixing of fish or fish by-product enzymes with acids, microorganisms and other enzymes [151]. Fish silage can be used in large-scale production because it is inexpensive and simple and has the main advantage of removing the smell and drainage problems of the industry. The disadvantages of fish silage include consumption at the same production place and high product volumes [152]. Fish silage and fish protein hydrolysates are commonly produced from the digestive organs of fish as well as the spleen and gonads [153]. Most nitrogen for the production of feed for pets and aquaculture is derived from these organs.

### 5.4. Fish Protein Hydrolysate

According to a recent survey, most food industries are using fish protein hydrolysates as an animal feed source [154]. Protein hydrolysates are significantly recovered from fish by-products because they contain high functional and nutritional properties. Several techniques are efficiently involved in the recovery of protein hydrolysates and many other natural products or high-value functional ingredients that can be used for animal feeding [155]. Additionally, biopeptides are a promising ingredient derived from fish by-products that are currently gaining increasing interest in the food industry [156]. Biopeptides are used for food fortification and exhibited various bioactivities such as antihypertensive, antioxidant, antimicrobial, anticholesterolemic, anticancer and antidiabetic activities [157]. Most fish protein hydrolysate sources are used for animal feed specifically those for poultry and swine. Protein hydrolysates in fish wastes are fermented under uncontrolled conditions, resulting in the production of high total volatile nitrogen contents and fish silage. Sheep fed fish silage gained more weight than those in the control feeding group [158]. Another protein ingredient is collagen, which can be a resultant product of fish-processing by-products.

### 5.5. Collagen

Collagen is considered to be the structural protein of connective tissues providing growth and support to bones, cartilage, skin, etc. This protein has numerous applications in the food, cosmetic, biomedical and pharmaceutical industries [159]. Collagen is widely applied in processed meat to improve the stability of products by increasing protein functionality through holding free water, used as a binding and extending agent in sausages and is involved in increasing the holding and absorption of water [160]. Collagen has some biomedical applications as a biopolymer to increase biocompatibility and decrease immunogenicity, producing fibres by cross-linking and self-aggregation. This protein is used in a bioprosthetic heart valve and wound dressing and it is also used for drug delivery by gel formulation with liposomes, transdermal delivery, gene delivery via nanoparticles and as a cell culture matrix [161].

Collagen is widely used in cosmetic products as an anti-ageing agent due to its useful biological effects [162]. Gelatine is the resultant product of collagen derived from fish processing by-products such as skin, bones, scales and internal organs of various fish species through thermal denaturation and partial hydrolysis. Gelatine has versatile industrial applications to enhance the consistency, stability and elasticity of dairy products, confectionery and fruit juices [163]. Gelatine alleviates phenol concentrations (tannins and anthocyanogens) and is presented for clarification and precipitation of fruit juices. At low melting temperatures, fish gelatine exhibited better aroma and flavour than those of bovine gelatine [164] and for diabetic patients, fish-based gelatine is used in food to increase protein levels and decrease carbohydrate levels [165]. Gelatine is used to make edible films that are heated for the manufacturing of edible containers for condiments and fried foods. In place of highly efficient catalysts, enzymes have great advantages in processing and are being used as biological catalysts to speed up biological reactions [166].

### 5.6. Enzymes

Many enzymes have been isolated from fish processing by-products and used as an alternative source of many enzymes. Viscera is a by-product of fish processing and is considered to be a good source of many enzymes such as protease, lipase, cellulase, and collagenase [167]. Enzymes or enzymatic peeling are used in the food, pharmaceutical and cosmeceutical industries and also serve as processing aids for various food products at low cost. Moreover, enzymes are used for the exclusion of undesirable parts of highly nutritious fish species [168]. There is a wide range of potential applications of fish enzymes in the dairy industry, especially for cheese production and various other industrial applications have also been reported [169].

#### 5.6.1. Proteases

Proteases are multifunctional enzymes that can be recovered from digestive enzymes (i.e., pepsin, chymotrypsin, elastase, gastricsin, trypsin, carboxylesterase and carboxypeptidase) of fish viscera and catalyse the hydrolytic degradation of proteins. Pepsin-rich sources are obtained from the stomach of several fish species such as cold- and warm-water fish, at optimal pH and temperature conditions [170]. Fish proteases and trypsin encode protein hydrolysate production from various fish species [171].

#### 5.6.2. Lipases

Lipases are isolated from various digestive organs of by-products such as the liver, stomach, and intestine. The liver shows higher lipase activity than that of other organs at optimal pH and temperature conditions of 8.0 °C and 50 °C, respectively, in the presence of ρ-nitrophenyl palmitate (ρ-NPP) substrate [172]. The potential uses of fish lipases have been employed for the alteration of foods, biodiesel production, flavour enhancement and production of structural lipids and delivered for hydrolysis of PUFAs for the enrichment of DHA and EPA in marine oils [173]. Fish lipases are extensively used in the dairy industry for flavour enhancement of dairy products and to inhibit the production of trans-fat in margarine. Lipases with antioxidants are used in the food and cosmetic industries to provide product stability [174] and in pharmaceuticals, lipases are applied as drugs to produce low serum cholesterol levels [175].

### 5.7. Minerals

It is likely that fish bones eliminated as by-products during fish processing contain significantly high amounts of mineral components presented as “bonefish”. Fish bones are generally employed as phosphorus and various other mineral forms to fulfil the calcium and phosphorus requirements of animal growth [176]. Fish bones are used as a fertilizer and as an animal feed extracted from the thermal processing of meat and bone meal during valorisation. Among the different parts of fish, cartilage and backbones consist of a high level of calcium phosphate, minerals and 30% proteins. Using various enzymes with a fish backbone helps to attain minerals and these minerals are utilized for feed formation with high functional properties [177].

### 5.8. Antioxidants

Several antioxidants and germicides are derived from by-products to increase food quality [178]. Many components contributed to better human health such as phenols, flavanols, and flavanol glycosides, when used in food compositions. These antioxidants have further applications in agriculture, mainly applied in pet feed formation and used as fertilizer after making a mixture of fish with carbon sources such as seashells and fish bones. These by-products are delivered as dry, liquid, fresh or frozen forms used for trap-making to catch fish [6].

## 6. Conclusions

The current review has interestingly reviewed the nutritional composition of fish and discussed the various applications of the utilization of fish processing by-products as well as preservative technologies to extend the shelf life of fish. Various processing technologies are actively involved to preserve the fish for a longer time. Enzymatic autolysis, microbial spoilage and chemical deterioration are three main factors involved in the spoilage of fish. It is necessary to control these factors by employing suitable techniques such as freezing, antimicrobials and antioxidants and super-chilling to ensure that the fish will be preserved for a long time. Low-temperature treatments can also be used efficiently to control microbial growth and enzymatic and non-enzymatic degradation processes. Several antimicrobial chemicals are effectively being applied to control microbial growth, whereas antioxidants are also used to control lipid oxidation. A broad range of technologies, including thermal and non-thermal treatments, are employed in industries for processing most economical fish products in such a way as to meet consumer demands with minimal quality damage. The main focus of these technologies is to extend the shelf life, enhance the nutritional value, extract high-value-added products and avoid any adverse effects on fish products with minimal processing and chemical preservative addition. The disposal of fish processing by-products has become a serious problem worldwide and is influenced by biological, technical, operational and economic factors. Environmental pollution caused by many by-products has received attention in order to utilize these by-products for various purposes. The main aim of by-product utilization is to overcome the disposal of by-products with the recovery of highly valuable products and other useful components from fish by-products. The nutritional value, preservative and processing technologies while utilization of fish processing by-products shall be discussed. Further, new studies should be focused on the optimization of processing technologies but also the evaluation of natural preservatives and by-product sources rich in all essential nutrients.

## 7. Future Perspectives

The future of fish with respect to preservation and processing depends upon the industrial utilization of new technologies and progressive management of the factors related to fish parameters such as quality, nutritional requirements, prolonged shelf life, new developmental products, freshness and high yield. In terms of future perspectives, more research is needed to understand the effects of processing and preservation parameters on fish and fish products and to make them more reliable and user-friendly for future development. To date, the discarding of various fish by-products is still considered a challenging factor that requires subsequent work and developmental technologies to recover highly valuable and new products for the long-term benefit of society and the environment.

## Figures and Tables

**Table 1:** Nutritional composition of fish muscle and their applications.

Nutrients	Percentage	Applications	Reference
Protein	15–24%	Potential source of animal protein, antioxidants and metabolic activities; improve muscle tissues and immunity; application in biotechnology and pharmaceutical.	[22]
Lipid	0.1–22%	Provide lipid-soluble vitamins (A and D) and essential omega-3s (PUFAs) absent in the body, lowering blood pressure and triglycerides in the blood; helps to reduce cardiovascular, childhood asthma, hypertension and Alzheimer’s disease.	[23]
- Docosahexaenoic acid (DHA)	6.1–10.3%	Helps to improve brain and neurodevelopment in children; involved in lipid metabolism and neural functioning and reduction in blood pressure and coronary heart disease.	[24]
Eicosapentaenoic acid (EPA)	3.7–4.5%	Protects against cardiovascular disease; involved in blood coagulation and aggregation of platelets; prevents dementia, atherosclerosis and rheumatoid arthritis.	[25]
Vitamins	0.1%	Improves growth and development of children; aids in bone, teeth and cell repair; prevents eyesight loss and blood coagulation; accelerates chemical processes in the body.	[26]
Minerals	1–2%	Have high bioavailability, easily absorbed by the body; helps in the synthesis of haemoglobin in RBCs and proper functioning of the thyroid gland.	[27]
-Calcium	0.5%	Mineralization and formation of bones; proper functioning of muscles and nervous system; involved in metabolic processes.	[28]
-Phosphorus	0.25%	Maintain teeth and bone structures; regulates acid–base equilibrium.	[28]

**Table 3:** Advantages and disadvantages of various non-thermal processing technologies.

Technologies	Advantages	Disadvantages	Storage Life	References
Pulsed electric field	Low energy consumption Short processing time Waste-free process	High initial investment Less efficient for spore inactivation Presence of bubbles effect uniformity Low economic	Improve tenderization and water holding capacity, less physiological effects due to partial disruption of cellular tissues, increase shelf life of meat	[107]
Ohmic treatment	Quick process Relatively uniform heating	High initial cost Relatively electrolytic effect		
Enzymatic treatment	More recovery yields Low contamination High selectivity rate	High enzymes cost Prolong processing time Low-efficiency rate	Increased shelf life through reducing oxidative spoilage, microbial activity, improve textural properties	[131]
Fluorescence spectroscopy	High data achievement rate Simple and more economic	Time consumption in sample preparation Not suitable for solid material detection Highly selective method	Improve protein functionality and conformational changes during protein denaturation	[112]
Nuclear magnetic resonance	High data evaluation Non-destructive and non-intrusive	High cost-effective Highly expensive equipment	Improve sensory properties, chemical composition, nutritional and physicochemical properties	[132]
Fermentation	More economical Environmentally friendly Useful for bioactive extraction Poor energy consumption	Slow process Recovery yield and quality effect by microorganism used	Reduce microbial proliferation, prevent foodborne pathogens, reduce microbial proliferation, therefore, extending shelf life	[133]
High hydrostatic pressure	Energy efficient High preservative quality Easy to commercialize Wide range of microorganism inactivation	Cost-effective Less efficient for spore’s inactivation Limited packaging facility	Prolonged shelf life up to 2 months at 2 ^o^C, reduce microbial load and food spoilage genera, improve quality	[134]
Fourier transform infrared spectroscopy (FTIR)	Rapid and reliable Sensitive to conformational changes under various conditions Independent of the physical condition of samples	Nonlinear problems of the curve High cost Strong IR absorbance of H_2_O	Monitored microbial spoilage, texture and colour attributes, authenticate freshness attributes	[114]
Raman spectroscopy	Required small size sample Less expensive instrumentations Non-destructive	Higher instrumental costs Stronger biological fluorescence interference Heat effect generated by the laser	Increase shelf life, improve protein and water contents, reduce microbial load	[135]
Near-infrared (NIR) spectroscopy	Rapid and non-destructive Non-contact and cost-effective	Accuracy depends on the reliability of the reference method Does not provide spatial information on the sample Contain unnecessary and redundant information	Reduce microbial spoilage, predict compositional changes, reduce foodborne pathogens	[136]
Visible near-infrared (VIS/NIR) spectroscopy	Non-contact Rapid Non-destructive	Non-independent requires samples with known analyte concentration Specular highlights and uneven illumination under varying sample surface	Reduce oxidation, optimize product quality, increase shelf life	[137]
Nuclear magnetic resonance/magnetic resonance imaging (NMR/MRI) spectroscopy	Cost-effective Non-destructive	Slow process High initial cost Expensive equipment	Food authentication, detect alteration and unwanted compounds.	[138]
Ultrafiltration	High energy efficiency Better quality permeates Continuous recovery	Time-consuming Expensive membranes	Extend shelf life, reduce disruption of cells, inhibit microbial spoilage	[139]
Supercritical fluid extraction	Environmentally friendly Rapidly penetrate in sample Mild processing conditions Low processing wastes	Complex equipment Required a high pressure Use of modifiers	Minimal disruption of tissue cells, improve quality, reduce bacterial count, improve shelf life	[140]

## Data Availability

Not applicable.
